# Genome-wide association study (GWAS) of ovarian cancer in Japanese predicted regulatory variants in 22q13.1

**DOI:** 10.1371/journal.pone.0209096

**Published:** 2018-12-17

**Authors:** Varalee Yodsurang, Yaqi Tang, Yukie Takahashi, Chizu Tanikawa, Yoichiro Kamatani, Atsushi Takahashi, Yukihide Momozawa, Nobuo Fuse, Junichi Sugawara, Atsushi Shimizu, Akimune Fukushima, Asahi Hishida, Norihiro Furusyo, Mariko Naito, Kenji Wakai, Taiki Yamaji, Norie Sawada, Motoki Iwasaki, Shoichiro Tsugane, Makoto Hirata, Yoshinori Murakami, Michiaki Kubo, Koichi Matsuda

**Affiliations:** 1 Laboratory of Clinical Genome Sequencing, Department of Computational Biology and Medical Sciences, Graduate School of Frontier Sciences, The University of Tokyo, Tokyo, Japan; 2 Institute of Pharmaceutical and Biological Sciences, University Claude Bernard Lyon 1, Lyon, France; 3 Department of Hematology, Teikyo University Chiba Medical Center, Chiba, Japan; 4 Laboratory of Genome Technology, Human Genome Center, Institute of Medical Science, The University of Tokyo, Tokyo, Japan; 5 RIKEN Center for Integrative Medical Sciences, Kanagawa, Japan; 6 Tohoku Medical Megabank Organization, Tohoku University, Sendai, Japan; 7 Iwate Tohoku Medical Megabank Organization, Iwate Medical University, Iwate, Japan; 8 Department of Preventive Medicine, Nagoya University Graduate School of Medicine, Nagoya, Japan; 9 Department of Environmental Medicine and Infectious Disease, Kyushu University, Fukuoka, Japan; 10 Department of Oral Epidemiology, Graduate School of Biomedical and Health Sciences, Hiroshima University, Hiroshima, Japan; 11 Division of Epidemiology, Center for Public Health Sciences, National Cancer Center, Tokyo, Japan; 12 Center for Public Health Sciences, National Cancer Center, Tokyo, Japan; 13 Division of Molecular Pathology, The Institute of Medical Science, The University of Tokyo, Tokyo, Japan; Memorial University of Newfoundland, CANADA

## Abstract

Genome-wide association studies (GWAS) have identified greater than 30 variants associated with ovarian cancer, but most of these variants were investigated in European populations. Here, we integrated GWAS and subsequent functional analyses to identify the genetic variants with potential regulatory effects. We conducted GWAS for ovarian cancer using 681 Japanese cases and 17,492 controls and found that rs137672 on 22q13.1 exhibited a strong association with a *P*-value of 1.05 × 10^−7^ and an odds ratio of 0.573 with a 95% confidence interval of 0.466–0.703. In addition, three previously reported SNPs, i.e., rs10088218, rs9870207 and rs1400482, were validated in the Japanese population (*P* < 0.05) with the same risk allele as noted in previous studies. Functional studies including regulatory feature analysis and electrophoretic mobility shift assay (EMSA) revealed two regulatory SNPs in 22q13.1, rs2072872 and rs6509, that affect the binding affinity to some nuclear proteins in ovarian cancer cells. The plausible regulatory proteins whose motifs could be affected by the allele changes of these two SNPs were also proposed. Moreover, the protective G allele of rs6509 was associated with a decreased *SYNGR1* expression level in normal ovarian tissues. Our findings elucidated the regulatory variants in 22q13.1 that are associated with ovarian cancer risk.

## Introduction

Ovarian cancer (OC) is one of the most common cancers among women worldwide [1]. The high mortality rate in ovarian cancer is due to late diagnosis resulting from the nonspecific nature of symptoms and lack of effective screening tools [2]. In the Japanese population, ovarian cancer exhibits the highest mortality rate compared with other gynecologic malignant tumors, and its prevalence has been increasing since 1975, although the main cause remains unclear [3]. Genome-wide association studies (GWAS) have identified greater than 30 variants associated with OC susceptibility. Most of these studies were conducted in European populations [4–10], and only two studies included Asian populations [11, 12]. Pathogenic variations in the *BRCA1* and *BRCA2* tumor suppressor genes responsible for most of hereditary breast and ovarian cancer syndromes [13] have been reported in numerous ethnic group including Japanese populations [14]. However, low-penetrance genetic variants still need to be elucidated, especially in Japanese populations.

To understand the functional consequences of cancer risk loci, post-GWAS analysis is performed, particularly with non-protein-coding variants. The goal is to uncover functional or causal SNPs that likely differ from associated SNPs obtained from GWAS. The systematic strategies for post-GWAS [15, 16] include the following: (1) targeting SNPs in linkage disequilibrium (LD) with the associated SNP; (2) determining mRNA expression levels of nearby genes that may be affected by the expression quantitative trait loci (eQTL); (3) characterization of gene regulatory regions; (4) identification of potential epigenetic mechanisms using tissue-specific data. In addition, (5) electrophoretic mobility shift assays (EMSA) are used to confirm the potential interaction between the tested variant and transcription factors (TF) [17]. Here, we performed a first population-based case–control GWAS in ethnical Japanese, and then selected the loci with the strongest associations for post-GWAS analyses.

## Materials and methods

### Patients and controls

All participants were ethnic Japanese women. The DNA samples of 681 ovarian cancer patients were stored in an automated DNA storage system; and 5μg of DNA samples (50 μl at a concentration of 100 ng/μl) were provided by Biobank Japan [18]. The 17,492 noncancer control female samples were obtained from four population-based cohorts: the JPHC (Japan Public Health Center)-based Prospective Study [19], the J-MICC (Japan Multi-Institutional Collaborative Cohort) study [20], ToMMo (Tohoku Medical Megabank Organization) and IMM (Iwate Tohoku Medical Megabank Organization) [21, 22]. The characteristics of each cohort are presented in Table 1; only the age of subjects was included in this analysis. All participating studies obtained written informed consents from all participants by following the protocols approved by their institutional ethical committees before enrollment. The consent procedure was approved by the ethical committees at each institute. This study was approved by the first ethics committee of Institute of Medical Science, The University of Tokyo (approval number of 29–74). We cannot access to any patient-level identifying information as part of the study.

**Table 1:** Characteristics of study population.

Sample type	Source	*N*	Age (mean ± SD)	Platform	Number of SNPs
Ovarian cancer cases	Biobank Japan	30	54.43±11.8	HumanExome_v10	247,870
				OmniExpress	730,525
		571	56.99±11.25	HumanExome_v11	242,901
				OmniExpress	730,525
		12	70.17±9.33	OmniExpressExome_v10	951,117
		68	58.03±12.57	OmniExpressExome_v12	964,193
	**Total**	**681**	**57.22 ± 11.51**		
Controls	1) JPHC	5,019	53.49 ± 7.80	OmniExpressExome_v12	964,193
	2) J-MICC	7,049	54.25 ± 9.41	OmniExpressExome_v12	964,193
	3) ToMMo	2,852	58.28 ± 11.95	OmniExpressExome_v12	964,193
	4) IMM	2,572	62.21 ± 10.03	OmniExpressExome_v12	964,193
	**Total**^a^	**17,492**	**55.86 ± 10.04**		

^a^ JPHC, Japan Public Health Center-based Prospective Study; J-MICC, Japan Multi-Institutional Collaborative Cohort study; ToMMo, Tohoku Medical Megabank Organization; IMM, Iwate Tohoku Medical Megabank Organization.

### GWAS genotyping and imputation

DNA genotyping and imputation were conducted at RIKEN Center for Integrative Medical Sciences in the previous studies for cases [23] and controls [24]. Genomic DNA samples were extracted from peripheral blood leukocytes using a standard method. All case and control samples were genotyped using the Illumina OmniExpress Exome or the OmniExpress+HumanExome BeadChip (Illumina Inc.). The type, version, and number of SNPs of genotyping platform used in each cohort were described in Table 1. A list of SNPs in each platform was obtained from Illumina. We select 925,436 common SNPs those were genotyped by any platforms for all samples. Allele calling algorithm used to compute the genotyping data was GenomeStudio V2011.1. A quality control was applied to the raw genotyping data to filter unqualified SNPs following the criteria as previously described [24]. We excluded SNPs that met the following criteria: minor allele frequency (MAF) < 0.01; Hardy-Weinberg equilibrium (HWE) *P*-value < 1 × 10^−6^; call rate < 0.99. We also exclude SNPs with a large allele frequency difference between the reference panel and the GWAS (> 0.16) as described previously [25]. After quality control, 498,990 SNPs were included for imputation analysis. Imputation of the ungenotyped SNPs was conducted with MaCH [26] and minimac [27] using the data from the JPT/CHS/CHB subjects and the 1000 genomes project phase 1 (release 16, March 2012) as a reference. Allele labels, as an effect or non-effect allele, and allele frequencies of imputed SNPs were obtained from minimac [27]. Post-imputation quality control was performed based on these following exclusion criteria: (1) MAF < 0.01; and (2) HWE *P*-value < 1 × 10^−6^. Finally, a total of 7,521,072 imputed SNPs were obtained for further analyses. The genotyping data is available at DNA DataBank of Japan (DDBJ) with an accession number: JGAD00000000123.

### Statistics

The statistical analyses were done for the SNPs that were common to different genotyping platforms used (Table 1) and whose genotype information was available for all cases and controls after imputations/implementation of quality control measures. The association between SNP and risk for developing ovarian cancer risk was investigated using logistic regression based on the first principal component (PC1) and the second principal component (PC2) as covariates [28]. The genetic inflation factor lambda (*λ*) was derived from *P*-values obtained using the Cochran–Armitage trend test for all the tested SNPs [29, 30]. The quantile-quantile plot was drawn using the R program. Odds ratios were calculated using the non-effect alleles as references. The effect size (beta) from the logistic model and the standard error for beta (SEbeta) were calculated using R program. The 95% confidence interval was calculated based on the following formula:

$$Lower\;limit\;of\;95\%\;CI=e^{beta\;-\;1.96\times SEbeta}$$

$$Upper\;limit\;of\;95\%\;CI=e^{beta+1.96\times SEbeta}$$

### SNP selection

We selected 201 candidate SNPs within 24 regions exhibiting a high association with ovarian cancer based on the following inclusion criteria: GWAS *P*-value < 1 × 10^−5^ and imputation quality score (Rsq) > 0.3 (S1 Table). Pairwise linkage disequilibrium (r^2^) between each SNP and lead SNP (the SNP with the lowest *P*-value in each region) in Japanese was obtained from Ensembl [31]. SNPs previously reported to be associated with ovarian cancer in published GWASs were obtained from GWAS catalog (https://www.ebi.ac.uk/gwas/). The data of reported SNPs, including risk allele and odd ratio, were retrieved from the original publications and further compared to the data of this Japanese dataset.

### Analysis of regulatory features

Thirty candidate SNPs at 22q13.1 were analyzed based on their location, epigenetic markers (i.e., H3K4Me1, H3K4Me3, and H3K27Ac) in ENCODE [32], Ensembl regulatory build indicating gene regulation, and TF binding data in Ensembl, ReMap [33], and JASPAR [34]. All data were visualized in the UCSC genome browser using track data hubs [35]. Regarding track settings in ReMap 2018, transcription regulators with peaks greater than 1.5 kb in size were retrieved from all public and ENCODE ChIP-seq data [33]. For JASPAR, we chose predicted binding sites with matching scores greater than 400 (*P*-value ≤ 10^−4^). The ovary-specific transcriptional regulations, including epigenome activity representing open chromatin and TF binding retrieved from ChIP-Seq data, were obtained from Ensembl. Regional plots were generated using LocusZoom (http://csg.sph.umich.edu/locuszoom).

The SNPs located in regulatory regions were further analyzed. The transcription factors reported in three databases and epigenome activity in ovaries were investigated. eQTL data of each SNP with nearby genes in normal ovarian tissues were obtained from Ensembl. The TF binding motifs containing SNP sequences were downloaded from HOCOMOCO [36], abstracting from ChIP-Seq datasets with quality A ratings.

### Electrophoretic mobility shift assay

SKOV3 cells were purchased from the American Type Culture Collection (ATCC). Cell culture was maintained using the depositor’s recommendations. Nuclear proteins from SKOV3 cells were extracted using NE-PER nuclear and cytoplasmic extraction reagents (Thermo Fisher Scientific) according to the manufacturer’s protocol. Protein concentrations were measured using a BCA protein assay (Thermo Fisher Scientific). EMSA was performed using DIG Gel Shift Kit, 2^nd^ Generation (Roche) following the manufacturer’s instruction with the additional step of re-annealing to eliminate the non-specific bands [37]. EMSA was performed two times separately, including screening for nine SNPs in 22q13.1 (S2 Fig) and confirming positive SNPs. The sequences of oligonucleotide probes are listed in S2 Table. In brief, 60 fmol of labeled probes containing SNP positions were hybridized with 5 mg of nuclear protein extract for 15 minutes at 20°C. The mixtures were then loaded into a 6% TBE gel, separated by electrophoresis at 4°C and transferred onto a nylon membrane. The membrane was then hybridized with anti-digoxigenin-AP antibody and developed by CSPD solution. The intensity of the shifted band was quantified using ImageJ software [38].

## Results

### GWAS of ovarian cancer in a Japanese population

**Fig 1:**
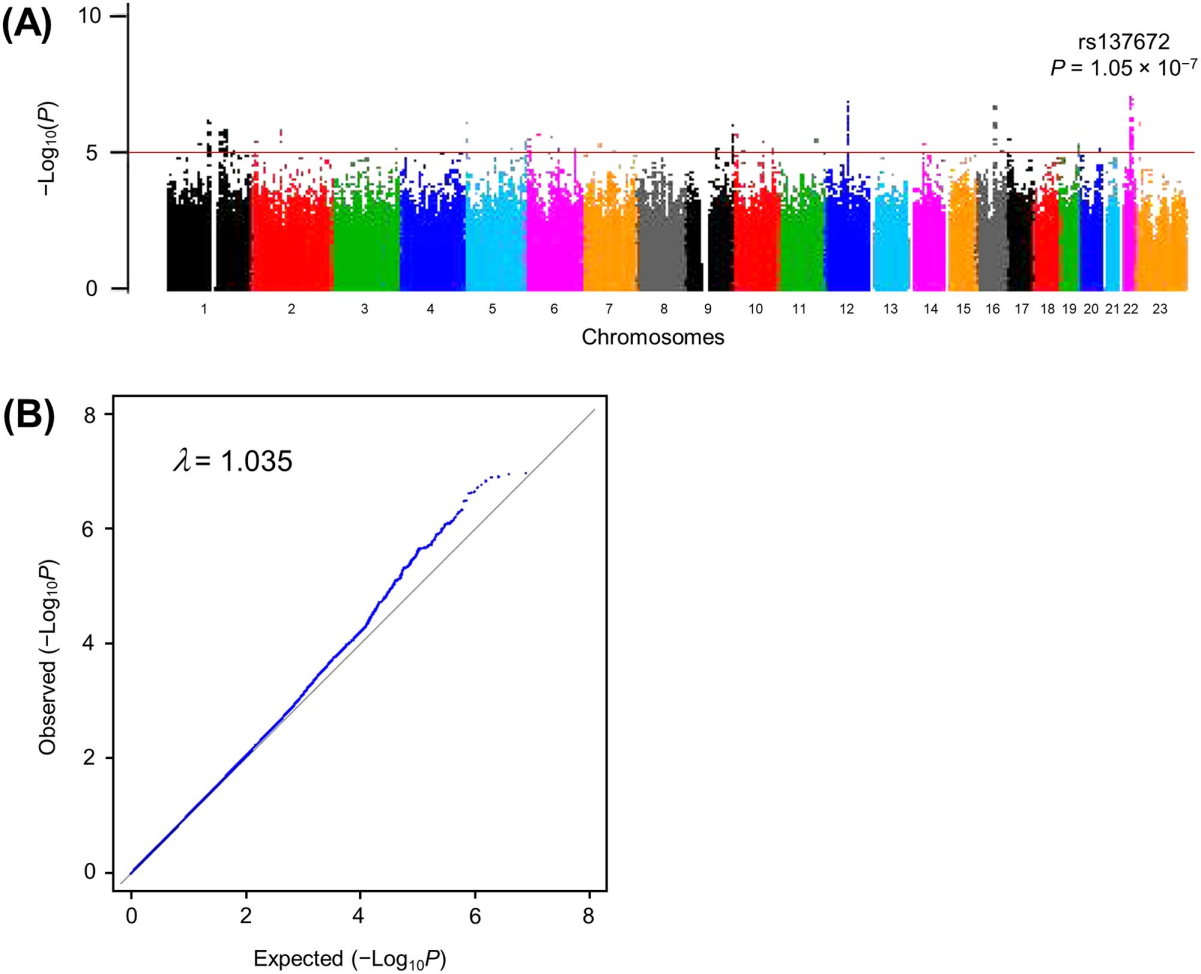
Genome-wide association results for ovarian cancer in a Japanese population. (A) Manhattan plot of 7,521,072 imputed SNPs on chromosomes 1 to 22. The red line indicates a threshold *P*-value of 1 × 10^−5^. Among 201 SNPs with *P*-values less than the threshold, rs137672 on chromosome 22 exhibited the lowest *P*-value (*P* = 1.05 × 10^−7^). (B) Quantile-quantile plot. The genomic inflation factor lambda (*λ*) was 1.035.

The DNA samples of 681 ovarian cancer patients and 17,492 cancer-free control females were genotyped by Illumina OmniExpress Exome or the OmniExpress+HumanExome BeadChip. The characteristics of each cohort are presented in Table 1. We conducted a standard quality control and genome-wide imputation analysis. The SNPs were excluded based on the following criteria: minor allele frequency (MAF) < 0.01; Hardy-Weinberg equilibrium *P*-value < 1 × 10^−6^; call rate < 0.99; GWAS allele frequency difference from the reference panel > 0.16. Consequently, we obtained the genotyping results of 7,521,072 imputed SNPs on autosomal chromosomes and analyzed their associations with OC risk (Fig 1A). The genomic inflation factor lambda (*λ*) was 1.035 (Fig 1B). We selected 201 candidate SNPs in 24 genomic regions demonstrating a suggestive association (*P*-value < 1 × 10^−5^). The most significant SNPs in each region, called lead SNPs, are presented in Table 2. Regional plots of 24 candidate loci are presented in S1 Fig. Among all candidates, rs137672 on 22q13.1 that is located in the upstream region of the *SYNGR1* gene (Synaptogyrin 1) exhibited the strongest association (*P* = 1.05 × 10^−7^; odds ratio of 0.573 with 95% confidence interval of 0.466–0.703). Detailed information of all 201 candidate SNPs are presented in S1 Table.

**Table 2:** Associations of lead SNPs in 24 regions meeting the criteria (*P* < 1 × 10^−5^ and Rsq > 0.3).

Locus	SNP	Eff/non allele^a^	Case freq^b^	Ctrl freq^b^	Rsq^c^	OR (95% CI)^d^	*P*-value	Gene	Relative location^e^
1p22.1	rs185345278	A/G	0.9664	0.9784	0.3344	0.348 (0.221–0.547)	4.86 × 10^−6^	*GCLM*	-23582
1p12	rs12031579	G/A	0.9177	0.9453	0.6395	0.548 (0.432–0.696)	7.88 × 10^−7^	*HAO2*	-60859
1q22	rs188625872	C/T	0.9885	0.9966	0.8794	0.266 (0.154–0.460)	2.01 × 10^−6^	*LMNA*	0
1q24.3	rs12117623	C/A	0.4070	0.4694	0.9943	0.762 (0.683–0.850)	1.46 × 10^−6^	*DNM3*	0
2p11.2	rs17027263	C/T	0.8437	0.8828	0.9998	0.693 (0.596–0.806)	1.69 × 10^−6^	*KDM3A*	0
3q27.3	rs6801612	A/G	0.7460	0.6830	0.9997	1.328 (1.174–1.503)	7.57 × 10^−6^	*RPL29P9*	1722
5p15.32	rs12658731	G/A	0.7030	0.6438	0.7678	1.405 (1.227–1.608)	8.57 × 10^−7^	*ADAMTS16*	0
5q31.2	rs147867139	A/G	0.9671	0.9825	0.7531	0.455 (0.322–0.642)	7.65 × 10^−6^	*CXXC5*	29634
5q35.3	rs60982503	G/A	0.7852	0.7345	0.9017	1.384 (1.204–1.591)	4.66 × 10^−6^	*CNOT6*	-9381
6p24.3	rs303051	A/G	0.5521	0.6053	0.9928	0.776 (0.696–0.867)	6.21 × 10^−6^	*TFAP2A*	0
6p21.31	rs56855829	C/T	0.9877	0.9964	0.8154	0.263 (0.151–0.459)	2.37 × 10^−6^	*FANCE*	0
6q16.1	rs4599655	A/C	0.8281	0.8635	0.9934	0.718 (0.621–0.829)	7.69 × 10^−6^	*FUT9*	68769
6q24.3	rs73589840	C/T	0.9452	0.9671	0.9537	0.573 (0.450–0.731)	7.42 × 10^−6^	*C6orf103*	0
7p12.3	rs181474944	C/T	0.9710	0.9850	0.6381	0.407 (0.276–0.600)	5.60 × 10^−6^	*MGC16075*	3731
7q21.13	rs76926936	T/G	0.9261	0.9483	0.4682	0.520 (0.390–0.694)	9.24 × 10^−6^	*ZNF804B*	361650
9q21.33	rs10117922	A/G	0.6249	0.6750	0.7793	0.749 (0.661–0.849)	7.35 × 10^−6^	*DAPK1*	0
9q34.3	rs10858374	T/C	0.8444	0.8894	0.9488	0.681 (0.583–0.795)	1.03 × 10^−6^	*C9orf62*	-95595
10q25.3	rs2615880	A/C	0.8385	0.8766	0.9776	0.713 (0.614–0.828)	8.30 × 10^−6^	*ATRNL1*	0
12q15	rs789336	C/T	0.6857	0.6082	0.9999	1.369 (1.217–1.540)	1.46 × 10^−7^	*C12orf28*	69976
16q12.2	rs145065165	G/A	0.9640	0.9777	0.4136	0.348 (0.233–0.519)	2.33 × 10^−7^	*RPL31P56*	5394
17p13.2	rs11870446	C/G	0.8360	0.8762	0.7774	0.674 (0.570–0.796)	3.37 × 10^−6^	*LOC339166*	0
19q13.43	rs12151036	G/T	0.9736	0.9881	0.9998	0.453 (0.322–0.636)	5.27 × 10^−6^	*ZNF274*	0
20q13.31	rs1884920	A/G	0.4534	0.4973	0.5171	0.711 (0.611–0.827)	8.29 × 10^−6^	*TFAP2C*	8727
22q13.1	rs137672	C/T	0.9045	0.9399	0.7961	0.573 (0.466–0.703)	1.05 × 10^−7^	*SYNGR1*	-8860

^a^ Effect allele/non-effect allele.

^b^ Effect allele frequency.

^c^ Rsq, imputation quality score.

^d^ OR, odd ratio (non-effect alleles were considered as references); 95% CI, 95% confidence interval.

^e^ Relative location, the distance from the transcription start site of the nearest gene to the SNP

### Associations of reported variants in a Japanese population

The imputed SNPs in this study were investigated whether they had been previously reported in published GWASs. First, we included reported SNPs that exhibited associations with OC susceptibility in any population, but not including the SNPs associated with specific OC subtypes or OC survival. Next, the reported SNPs were searched in this GWAS and found that 34 SNPs, reported in nine studies [4–12], passed the quality control and could be evaluated in the Japanese dataset (Table 3). Among nine studies, two included Asian populations [11, 12]. Chen et al. conducted GWAS with Han Chinese subjects; whereas Pharoah et al. pooled GWAS from European countries and confirmed the associations in various European and Asian populations. A concordance between studies was investigated based on risk alleles and odd ratios. We compared the associations of seven SNPs reported in both Asian and European populations in previous studies and found the concordances between those ethnicities. Among 34 reported SNPs (23 loci) with Japanese data, 25 SNPs (18 loci) exhibited the same risk allele as reported in previous studies; though only three SNPs, i.e., rs9870207, rs1400482, and rs10088218, exhibited significant associations (*P* < 0.05).

**Table 3:** Associations of the previously reported SNPs in the study population.

Locus	SNP	Eff/non allele^a^	GWAS results in this study (Japanese)					Gene	Previous study	
			Case freq^b^	Ctrl freq^b^	*P*-value	Rsq^c^	OR (95% CI)^d^		Concordance with this study	PMID (population)^e^
2q13	rs2165109	A/C	0.5403	0.5183	1.72 × 10^−1^	0.9929	1.079 (0.967–1.204)	*ACOXL*	opposite	28346442 (EU)
2q13	rs17041869	A/G	0.7439	0.7474	8.50 × 10^−1^	0.9987	0.988 (0.873–1.118)	*BCL2L11*	opposite	28346442 (EU)
2q14.1	rs752590	A/G	0.8554	0.8459	2.60 × 10^−1^	0.8992	1.098 (0.934–1.293)	*PAX8*	same	26075790 (EU)
2q31.1	rs711830	G/A	0.7832	0.7938	7.39 × 10^−1^	0.9972	0.978 (0.858–1.116)	*HOXD3*	same	28346442 (EU)
2q31.1	rs2072590	C/A	0.7830	0.7935	7.52 × 10^−1^	0.9970	0.979 (0.859–1.117)	*LOC401022*	same	20852632 (EU) 23535730 (EU+AS)
2q31.1	rs6755777	G/T	0.7828	0.7931	7.68 × 10^−1^	0.9982	0.980 (0.860–1.118)	*LOC401022*	same	28346442 (EU)
3q25.31	rs2665390	T/C	0.9934	0.9901	1.39 × 10^−1^	0.9994	1.655 (0.850–3.223)	*TIPARP*	same	20852632 (EU) 25134534 (AS)
3q28	rs9870207	A/G	0.5387	0.5034	**3.53 × 10^−2^**	0.9994	1.124 (1.007–1.255)	*LOC100131685*	same	28346442 (EU)
4q32.3	rs13113999	T/G	0.9397	0.9439	2.94 × 10^−1^	0.7344	0.867 (0.664–1.132)	*TLL1*	opposite	28346442 (EU)
5p15.33	rs10069690	C/T	0.7485	0.7550	2.22 × 10^−1^	0.9847	0.925 (0.816–1.049)	*TERT*	same	25581431 (EU) 25134534 (AS)
5p15.33	rs7705526	C/A	0.6226	0.6361	1.27 × 10^−1^	0.8361	0.909 (0.805–1.027)	*TERT*	same	28346442 (EU)
7p12.1	rs2190503	G/A	0.8747	0.8781	1.91 × 10^−1^	0.9948	0.895 (0.760–1.056)	*GRB10*	same	24190013 (EU)
7p12.1	rs6593140	T/C	0.8869	0.8875	3.15 × 10^−1^	0.9998	0.916 (0.771–1.088)	*GRB10*	same	24190013 (EU)
7p12.1	rs2329554	G/A	0.6888	0.6946	4.71 × 10^−1^	0.9890	0.957 (0.852–1.077)	*GRB10*	same	24190013 (EU)
8q24.21	rs9886651	A/G	0.7318	0.7160	4.96 × 10^−1^	0.9823	1.044 (0.923–1.181)	*PVT1*	opposite	28346442 (EU)
8q24.21	rs1400482	G/A	0.9897	0.9830	**4.94 × 10^−2^**	0.9867	1.709 (1.001–2.919)	*MIR1208*	same	28346442 (EU)
8q24.21	rs10088218	G/A	0.9897	0.9829	**4.73 × 10^−2^**	0.9988	1.713 (1.007–2.913)	*MIR1208*	same	20852632 (EU) 25134534 (AS)
9p22.2	rs3814113	T/C	0.7525	0.7519	8.30 × 10^−1^	0.9975	1.014 (0.895–1.150)	*BNC2*	same	19648919 (EU) 25134534 (AS)
9q22.33	rs1413299	T/G	0.7249	0.7326	7.96 × 10^−1^	0.9988	0.984 (0.872–1.111)	*COL15A1*	same	25134534 (AS)
9q34.2	rs633862	T/C	0.5470	0.5265	3.05 × 10^−1^	1.0000	1.058 (0.950–1.179)	*ABO*	same	25134534 (AS)
10p11.21	rs1192691	G/T	0.4723	0.4746	4.02 × 10^−1^	0.9902	0.954 (0.855–1.065)	*LOC389948*	opposite	25134534 (AS)
12q14.2	rs11175194	G/A	0.6417	0.6377	6.76 × 10^−1^	0.9998	1.024 (0.914–1.148)	*SRGAP1*	same	25134534 (AS)
12q22	rs11108890	C/A	0.8847	0.8933	9.89 × 10^−2^	0.9994	0.867 (0.731–1.028)	*TRNAQ46P*	same	24190013 (EU)
13q14.2	rs970651	G/A	0.7590	0.7533	8.57 × 10^−1^	0.9844	1.012 (0.893–1.147)	*SUCLA2*	opposite	24190013 (EU)
14q24.1	rs17106154	T/C	0.6988	0.6817	2.61 × 10^−1^	0.9093	1.074 (0.949–1.215)	*RPL12P7*	opposite	24190013 (EU)
17q12	rs7405776	G/A	0.6906	0.6873	8.46 × 10^−1^	0.6209	1.015 (0.875–1.178)	*HNF1B*	same	28346442 (EU)
17q12	rs757210	C/T	0.6854	0.6821	8.57 × 10^−1^	0.6656	1.013 (0.878–1.169)	*HNF1B*	opposite	25581431 (EU) 23535730 (EU+AS)
17q12	rs11651755	T/C	0.6799	0.6779	9.76 × 10^−1^	0.9990	1.002 (0.891–1.127)	*HNF1B*	same	28346442 (EU)
17q21.31	rs183211	A/G	0.7048	0.7051	7.30 × 10^−1^	0.8396	1.023 (0.899–1.165)	*NSF*	same	25581431 (EU)
17q21.32	rs9303542	A/G	0.8094	0.8094	3.63 × 10^−1^	0.9788	0.937 (0.815–1.077)	*SKAP1*	same	25581431 (EU) 25134534 (AS)
19p13.11	rs2363956	T/G	0.6970	0.6894	2.52 × 10^−1^	0.9995	1.071 (0.953–1.205)	*ANKLE1*	opposite	20852633 (EU)
19p13.11	rs1469713	A/G	0.7357	0.7374	9.17 × 10^−1^	0.9890	0.993 (0.878–1.124)	*GATAD2A*	same	28346442 (EU)
22q12.1	rs6005807	C/T	0.9751	0.9685	1.32 × 10^−1^	0.9183	1.324 (0.920–1.907)	*TTC28*	same	28346442 (EU)
22q12.2	rs9609538	T/C	0.8122	0.8025	2.06 × 10^−1^	0.9986	1.094 (0.952–1.258)	*BPIFC*	same	24190013 (EU)

^a^ Effect allele/non-effect allele.

^b^ Effect allele frequency.

^c^ Rsq, imputation quality score.

^d^ OR, odd ratio (non-effect alleles were considered as references); 95% CI, 95% confidence interval.

^e^ EU, European; AS, Asian.

### Analysis of regulatory features

**Fig 2:**
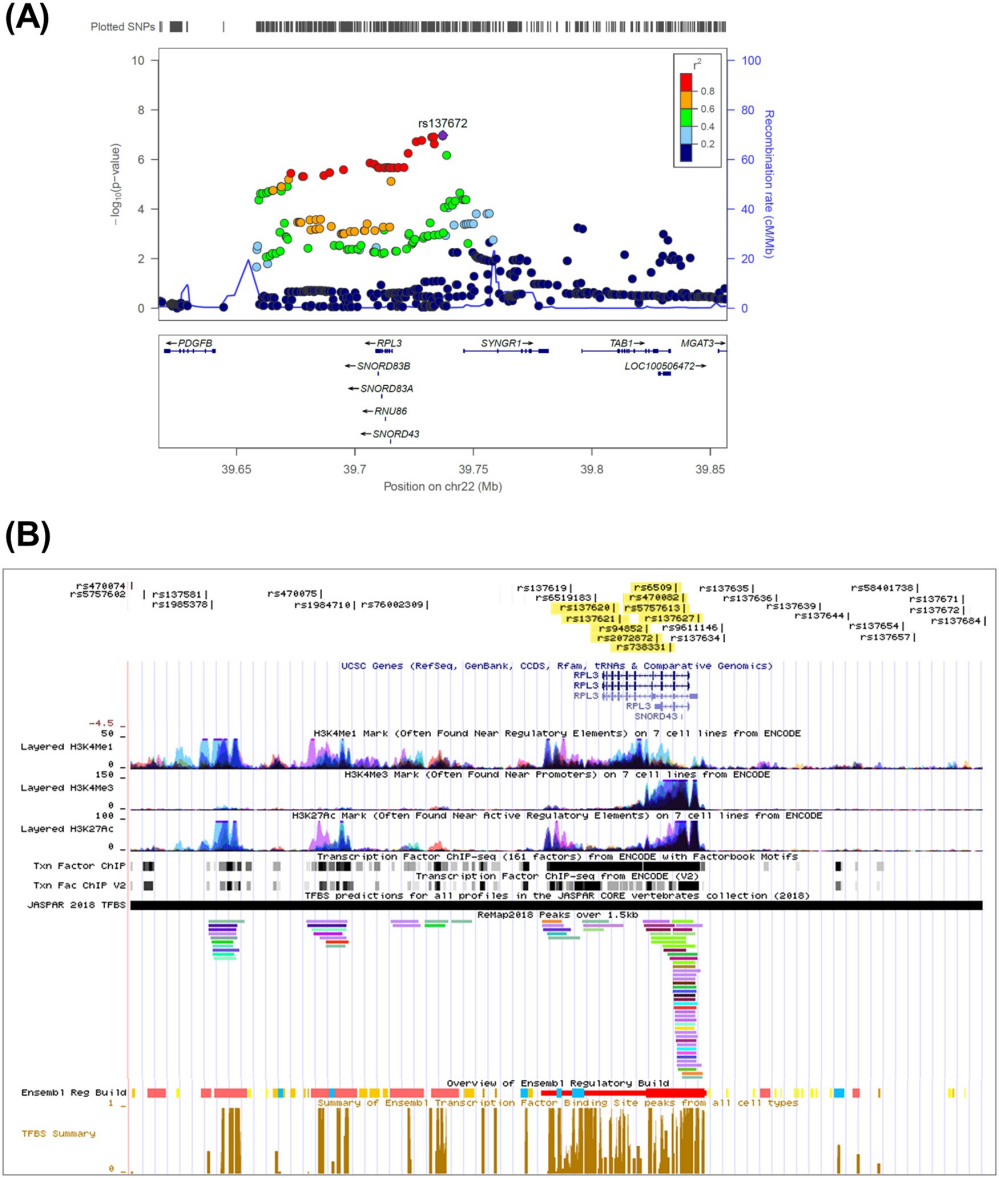
Regulatory feature analysis of 30 candidate SNPs at 22q13.1. (A) The regional plot of 22q13.1, the most associated loci in a Japanese population, with an enlarged view of 30 candidate SNPs meeting the criteria (*P* < 1 × 10^−5^ and Rsq > 0.3). The SNPs surrounding the lead SNP (rs137672) are color-coded to reflect their correlation as indicated. Pairwise r^2^ values are obtained from 1000 Genomes East Asian data (March 2012 release). Genes, the position of exons and the direction of transcription obtained from the UCSC genome browser. (B). Thirty SNPs were marked on chromosome 22 (chr22:39,671,929–39,738,825; GRCh37/hg19) based on their positions. Nine yellow-highlighted SNPs, variant located in regulatory regions in 22q13.1. The regulatory features included 1) epigenetic markers, i.e., H3K4Me1, H3K4Me3, and H3K27Ac; 2) Ensembl regulatory build representing regions involved in gene regulation; 3) transcriptional regulation binding data.

From GWAS results, we selected the most strongly associated loci (22q13.1) in a Japanese population, including 30 candidate SNPs that passed the criteria (*P* < 1 × 10^−5^ and Rsq > 0.3) for post-GWAS analyses. The enlarged view of the regional plot for these 30 candidate SNPs is presented in Fig 2A. We analyzed the following regulatory features (Fig 2B): 1) epigenetic markers indicating an active promotor or enhancer region, i.e., H3K4Me1, H3K4Me3, and H3K27Ac; 2) regulatory build indicating regions that are likely to be involved in gene regulation; 3) transcriptional regulation data. The results demonstrated that nine SNPs were located in regions with positive epigenetic markers and transcription factor binding sites based on ReMap and ENCODE (Fig 2B); however, these regions did not include the lead SNP rs137672 or seven SNPs with absolute linkage disequilibrium with the lead SNP (r^2^ = 1) (S1 Table). Among nine SNPs in regulatory regions in 22q13.1, only rs6509 was located in the protein-coding region on exon 2 of the *RPL3* gene. However, this variant was a synonymous SNP that did not affect the protein sequence. Moreover, five SNPs were located in predicted promotor regions active in ovary cells reported by Ensembl, i.e., rs738331, rs6509, rs470082, rs5757613, and rs137627 (Table 4). The eQTL data demonstrated significant associations (*P* < 0.05) with *SYNGR1* level for six SNPs, i.e., rs137620, rs137621, rs94852, rs6509, rs470082, and rs137627. Only rs137627 was also associated with *PDGFB* level (Table 4). Noteworthy, the eQTL effect size of all nine SNPs revealed that the trend of association with *RPL3* was in the opposite direction to that with *PDGFB* and *SYNGR1*.

**Table 4:** Transcriptional regulations and eQTL analysis of the nine SNPs in regulatory regions in 22q13.1.

SNP	r^2^ to rs6509^a^	Transcriptional regulations^b^			eQTL in ovary						
		ReMap	Ensembl (data in ovary)		Ref/alt allele^c^	*PDGFB*		*RPL3*		*SYNGR1*	
		TF	Epigenome activity	TF		*P*-value	Beta^d^	*P*-value	Beta^d^	*P*-value	Beta^d^
rs137620	1.000	atf2	N/A	-	T/C	0.102	0.18	0.777	-0.04	0.025	0.19
rs137621	1.000	atf2	N/A	-	G/A	0.079	0.19	0.784	-0.04	0.015	0.21
rs94852	1.000	sin3a, bcl6	N/A	-	T/C	0.119	0.16	0.42	-0.12	0.023	0.19
rs2072872	1.000	sin3a, bcl6, mllt1, smad5, chd1	N/A	-	A/G	0.14	-0.2	0.368	0.17	0.297	-0.11
rs738331	1.000	sin3a, bcl6, mllt1, smad5, chd1, max	Active promotor (G allele)	Cjun, Gabp, Jund, FOSL2, Egr1, CTCF, Yy1, JUN::FOS, SP1	A/G	0.14	-0.2	0.368	0.17	0.298	-0.11
rs6509	1.000	bcl6, chd1, mllt1, max, taf3	Active promotor (T allele)	Yy1, Jund, SP1, CTCF, JUN::FOS, Cjun, FOSL2, Gabp, Egr1	C/T	0.065	0.2	0.648	-0.07	0.005	0.23
rs470082	1.000	chd1, mllt1, max, taf3, bclaf1, smad5, bcor, brd4, atf2, mxi1, cdk8, myc, taf1, smc3, sin3a, rad21, cbfb, bcl6, chd8, phf8, med1	Active promotor (T allele)	SP1, Yy1, JUN::FOS, Egr1, FOSL2, Gabp, Jund, Cjun, CTCF	C/T	0.091	0.19	0.379	-0.13	0.003	0.25
rs5757613	0.543	chd1, mllt1, max, taf3, bclaf1, smad5, bcor, brd4, atf2, mxi1, cdk8, myc, taf1, smc3, sin3a, rad21, cbfb, bcl6, chd8, phf8, med1	Active promotor (A allele)	Cjun, Jund, Gabp, Egr1, FOSL2, CTCF, JUN::FOS, Yy1, SP1	G/A	0.116	-0.22	0.164	0.26	0.171	-0.15
rs137627	1.000	chd1, max, taf3, smad5, bcor, brd4, atf2, mxi1, cdk8, taf1, smc3, sin3a, rad21, bcl6, chd8, phf8, med1, ctcf, stag1	Active promotor (C and A alleles)	JUN::FOS, FOSL2, SP1, Cjun, Gabp, Egr1, CTCF, Yy1, Jund	G/A	0.049	0.22	0.383	-0.13	0.002	0.27

^a^ Pairwise linkage disequilibrium (r^2^) to rs6509 in Japanese.

^b^ Transcriptional regulations in ReMap and Ensembl; TF, transcription factor.

^c^ Reference/alternative allele.

^d^ Beta, effect size representing the effect of alternative allele to the gene expression; (value > 0), increased expression; value < 0, decreased expression.

### Allele specific binding of nuclear proteins to rs2072872 and rs6509

**Fig 3:**
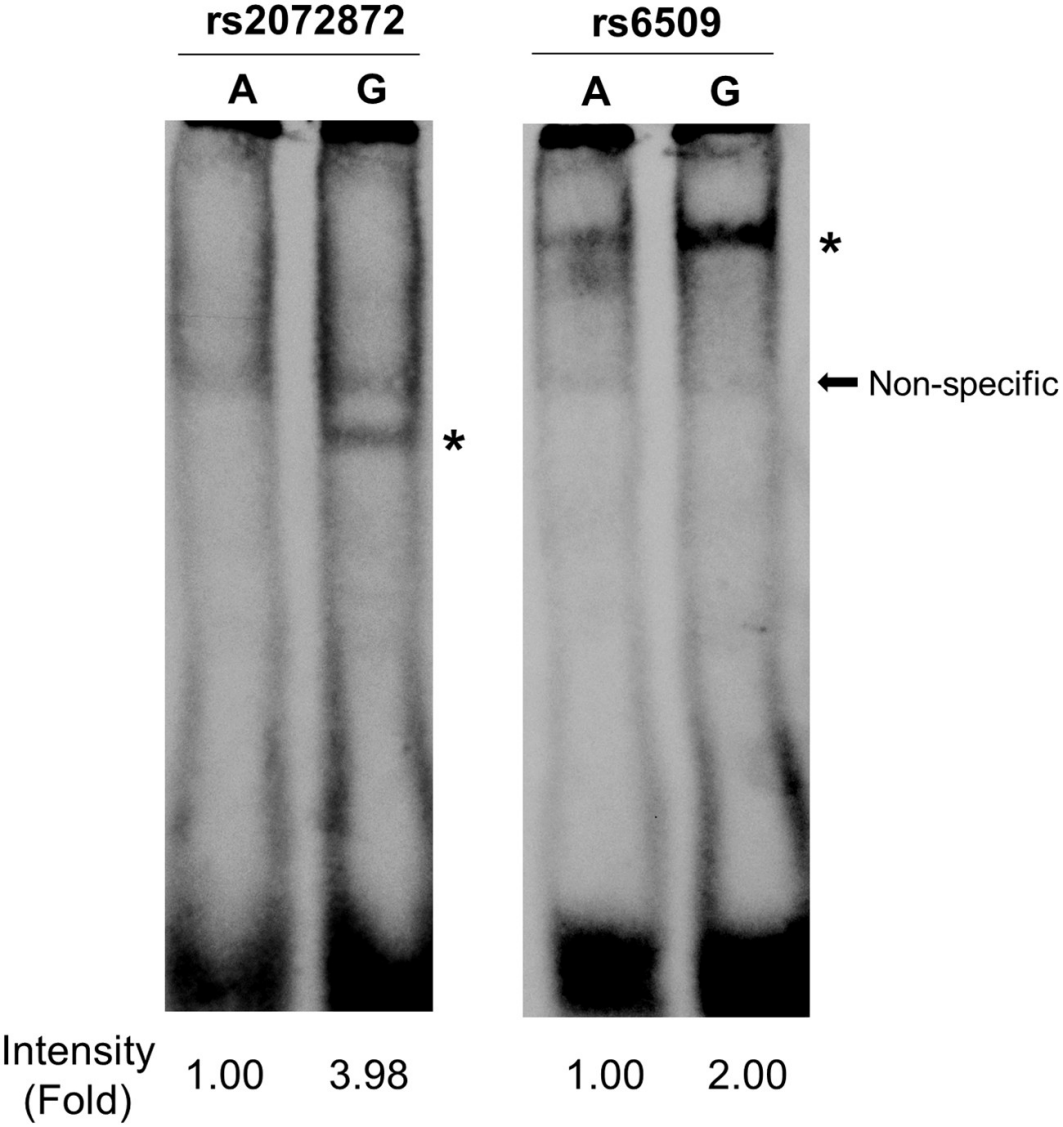
Allele-specific binding of nuclear proteins to rs2072872 and rs6509. EMSA using 31-bp labeled oligonucleotide probes flanking each SNP (SNP ± 15 bp). Sequences of oligonucleotide probes are listed in S2 Table. The shifted band indicated the interaction between nuclear protein extracted from SKOV3 cells and probes containing the SNP allele as indicated, i.e., A or G allele. The star indicates specific binding to the G allele of each SNP. Arrow indicates non-specific interaction found in every sample. The intensity of a shifted band was quantified based on the fold-change of the G allele with respect to the A allele using ImageJ software.

**Fig 4:**
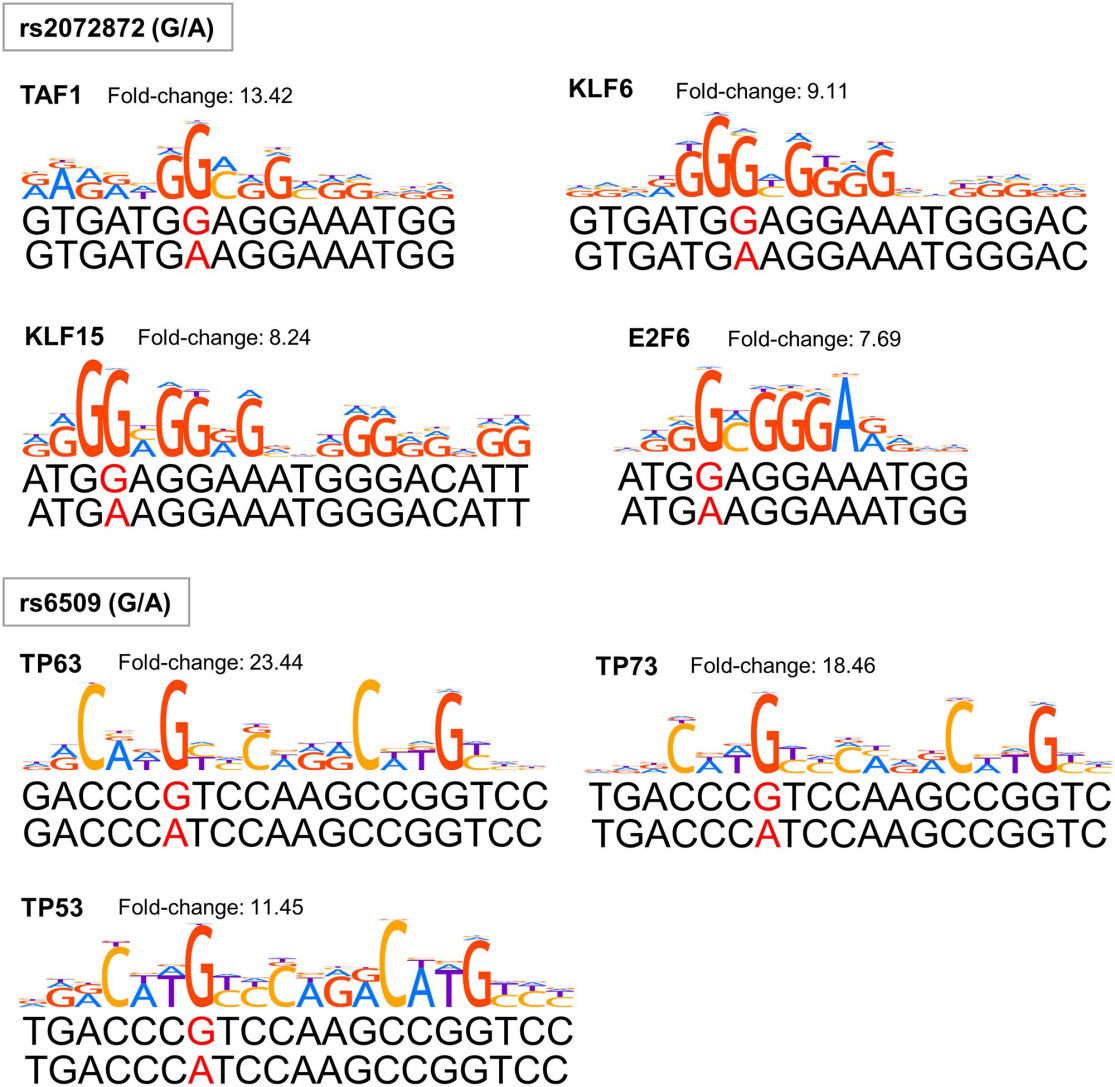
The predicted transcription factor motifs containing rs2072872 and rs6509. The SNP with flanking sequences (SNP ± 25 nucleotides) was searched for transcription factor binding site motifs using HOCOMOCO-11 collection (http://opera.autosome.ru) with a p-value cutoff = 0.0005 and fold-change cutoff = 4.0. The motifs built from ChIP-Seq data with quality A demonstrating high affinity (indicated by fold-change of G/A) for G allele are included. The SNP position is labeled in red.

To investigate whether the genetic variations in 22q13.1 affect the binding affinity of some transcription factors, we performed the electrophoretic mobility shift assays using the nine candidate SNPs. We examined the binding of nuclear proteins extracted from SKOV3 human ovarian cancer cells and a labeled oligonucleotide corresponding to each allele of the candidate SNPs. Among the three SNPs that exhibited allele-specific binding in the screening (S2 Fig), rs2072872 and rs6509 showed consistent results in the confirmation step (Fig 3). The oligonucleotides corresponding to G alleles of these two SNPs exhibited stronger binding affinity to nuclear proteins compared with A alleles (S2 Fig and Fig 3). Several transcription factors, including KLF6 and TP53, are predicted to bind DNA fragments containing these SNPs with different affinities as shown in Fig 4.

## Discussion

This is the first GWAS for ovarian cancer using Japanese case–control samples. Furthermore, the functional analyses were carried out following a GWAS to distinguish functional from non-functional risk SNPs. Novel 201 SNPs in 24 loci exhibited an association with ovarian cancer susceptibility with *P*-value less than 1 × 10^−5^ (S1 Table). Among all candidates, rs137672 in the upstream region of *SYNGR1* gene at 22q13.1 was the most associated variant (*P* = 1.05 × 10^−7^). Given the relatively small number of patients, if compared to previously published GWASs [4, 5, 11], no SNPs with a significant GWAS *P*-value (< 5 × 10^−8^) was observed in this study. Indeed, the incidence of ovarian cancer in Japanese population was lower (age-standardized rate = 8.4 per 100,000 persons/year in 2012) than that reported in European population that exhibited the highest incidence in central and eastern Europe (age-standardized rate = 11.4 per 100,000 persons/year in 2012) [1]. However, the prevalence of pathogenic variants in *BRCA1/2* seems comparable across diverse ethnicities, including European and Asian women [39], suggesting that other risk or protective factors still need to be identified. In the present study, we verified the significant associations (*P* < 0.05) of three previously reported SNPs in a Japanese population; all SNPs exhibited similar associations with other ethnicities (Table 3). Among these variants, the association of rs10088218 was previously reported in an Asian population (Han Chinese) [12], whereas rs9870207 and rs1400482 were investigated in Asian for the first time in this study.

The regulatory feature analysis of 30 SNPs (*P* < 1 × 10^−5^) with the strongest associations (22q13.1) unveiled nine candidate functional SNPs that exhibited interactions with some transcription factors based on ChIP-Seq databases and positive histone marks associated with active promotor or enhancer based on ENCODE (Fig 2). We subsequently analyzed nine candidate causal SNPs using EMSA and identified two regulatory SNPs, rs2072872 and rs6509, that affected the binding affinity to nuclear proteins from ovarian cancer cells (Fig 3). In GWAS, the association results of rs2072872 (G/A) were OR (95% CI) = 0.612 (0.501–0.750) with *P* = 2.08 × 10^−6^, and the results for rs6509 (C/T or G/A) were OR (95% CI) = 0.613 (0.501–0.750) with *P* = 2.16 × 10^−6^ (S1 Table). The distances between the SNP with the strongest association with OC risk (rs137672) and the regulatory SNPs, rs2072872 and rs6509, were 23.97 kilobase and 22.60 kilobase, respectively. The pairwise r^2^ of these two SNPs to the SNP rs137672 was 0.85 in Japanese (S1 Table), suggesting that the strong association of rs137672 may be influenced by the two regulatory SNP. The eQTL results demonstrated that the T (A) allele of rs6509 was associated with increased *SYNGR1* levels (effect size = 0.23, *P* = 0.005) (Table 4). Given that these two SNP are in complete LD (r^2^ = 1, both SNP’s G alleles are correlated), the post-GWAS results can possibly predict the regulation of transcription factor(s) that synergistically regulate(s) the decreased expression of *SYNGR1* through binding to G allele of rs6509 and rs2072872 simultaneously. However, *in vivo* experiments are essential to verify that either or both of rs6509 and rs2072872 have the regulatory functions. GWAS identified G alleles of both SNPs as being associated with reduced OC risk. Our finding suggested that *SYNGR1* and higher level of *SYNGRI* expression may plausibly increase ovarian cancer risk.

The *RPL3* gene encodes a ribosomal protein L3 that plays an essential role in the initial step of protein translation [40, 41]. Moreover, RPL3 is involved in modulation of cell cycle and apoptosis pathways [42] and serves as a target of Omacetaxine, an anticancer drug used for chronic myeloid leukemia [43]. *RPL3* mRNA expression is extraordinarily high in ovarian tissue compared with other organs [44], highlighting some important functions that should be investigated. Although SNPs at 22q13.1 were not associated with *RPL3* expression based on eQTL data, further studies should focus on functional roles of these SNPs and *RPL3* in ovarian cancer risk. In addition, the role of *SYNGR1* in ovaries should be clarified.

In conclusion, we utilized GWAS and post-GWAS analyses to identify regulatory genetic variants that were predicted to function as transcriptional regulators, without causing amino acid changes. Although further replication studies are essential, our results elucidated the important role of genetic variations in the development of OC among the Japanese population.

## Supporting information

S1 Table Associations of 201 candidate SNPs meeting the criteria (*P* < 1 × 10^−5^ and Rsq > 0.3). (XLSX)

S2 Table List of oligonucleotide probes used in EMSA. (XLSX)

S1 Fig Regional plots of 24 candidate loci reported in this study. (PPTX)

S2 Fig EMSA results of the nine SNPs in regulatory regions in 22q13.1. (TIF)

## Abbreviations

ASR: age-standardized rate
CI: confidence interval
EMSA: electrophoretic mobility shift assay
eQTL: expression quantitative trait loci
GWAS: genome-wide association studies
HWE: Hardy-Weinberg equilibrium
LD: linkage disequilibrium
MAF: minor allele frequency
OC: ovarian cancer
OR: odd ratio
TF: transcription factor
