# Supplementary material for: Genome-wide association study (GWAS) of ovarian cancer in Japanese predicted regulatory variants in 22q13.1
Source: PLoS One. 2018 Dec 17;13(12):e0209096. doi: 10.1371/journal.pone.0209096 (PMC6296504; doi:10.1371/journal.pone.0209096)
Supplement: S1 Fig — (PPTX) [file pone.0209096.s003.pptx]

## Slide 1
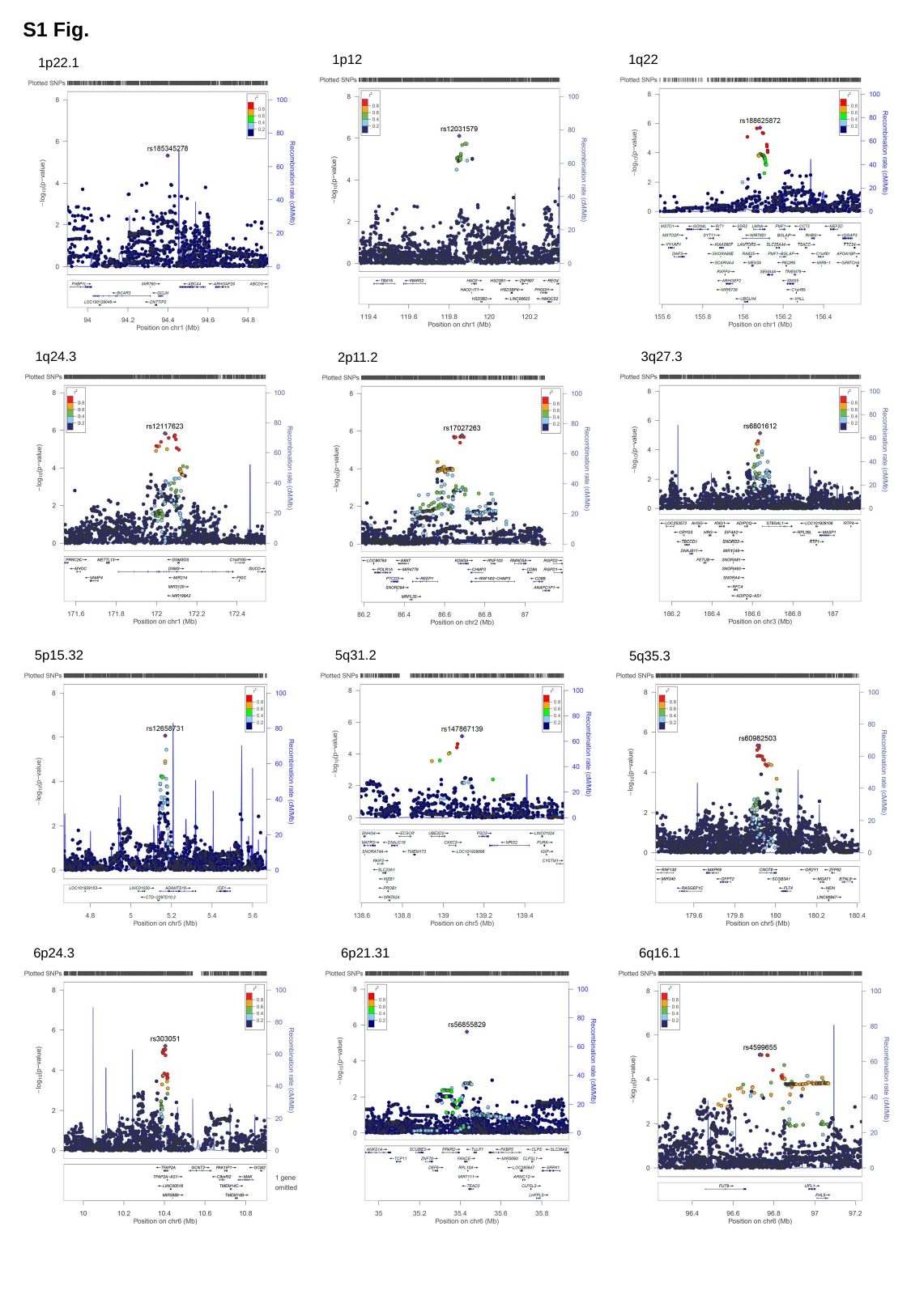

S1 Fig.
1p12
1q22
1p22.1
1q24.3
3q27.3
2p11.2
5q31.2
5p15.32
5q35.3
6p24.3
6p21.31
6q16.1

## Slide 2
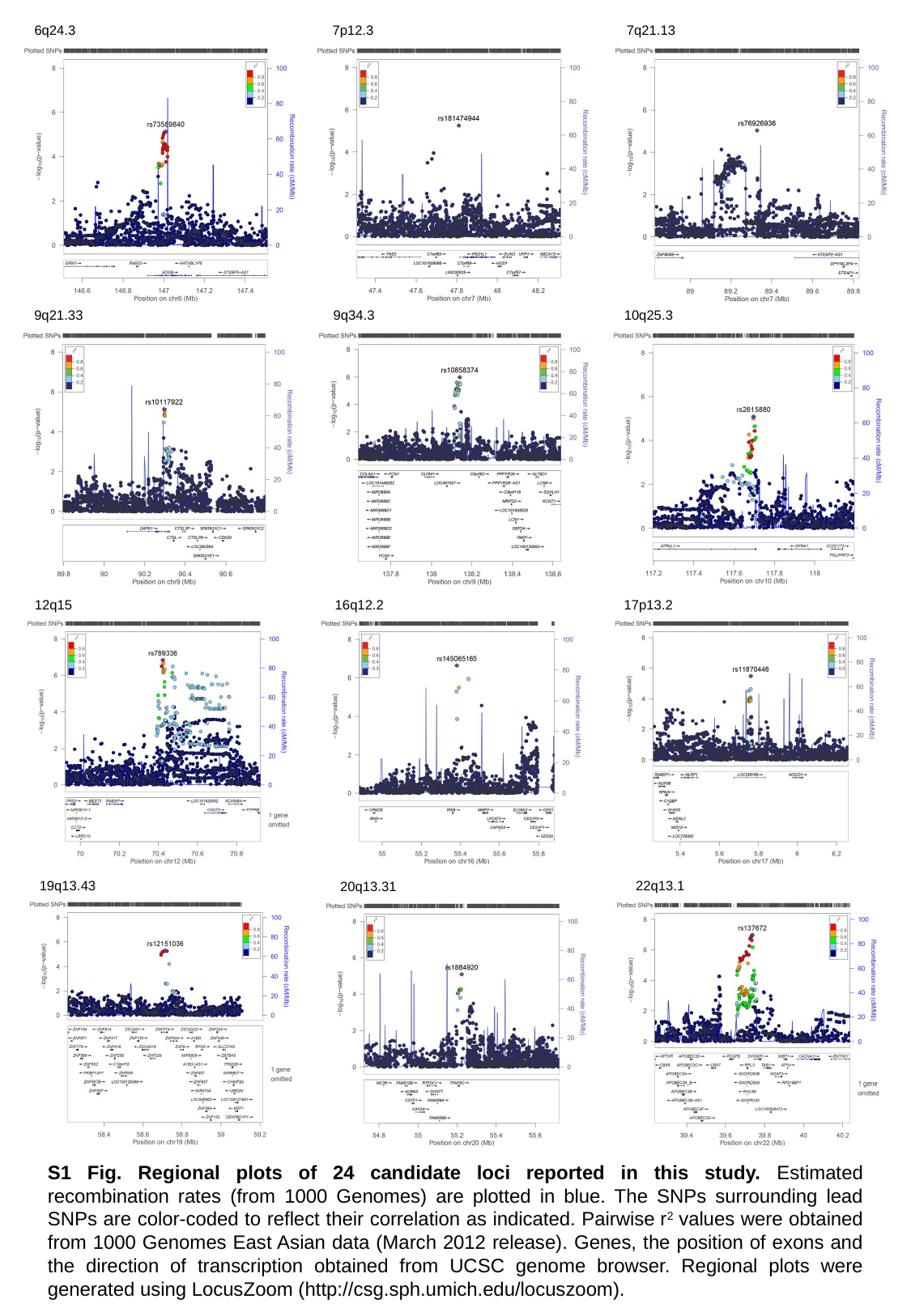

6q24.3
7p12.3
7q21.13
9q21.33
9q34.3
10q25.3
12q15
16q12.2
17p13.2
19q13.43
22q13.1
20q13.31
S1 Fig. Regional plots of 24 candidate loci reported in this study. Estimated recombination rates (from 1000 Genomes) are plotted in blue. The SNPs surrounding lead SNPs are color-coded to reflect their correlation as indicated. Pairwise r2 values were obtained from 1000 Genomes East Asian data (March 2012 release). Genes, the position of exons and the direction of transcription obtained from UCSC genome browser. Regional plots were generated using LocusZoom (http://csg.sph.umich.edu/locuszoom).
