# Supplementary figures and images for: Genome-wide association study (GWAS) of ovarian cancer in Japanese predicted regulatory variants in 22q13.1
Source: PLoS One. 2018 Dec 17;13(12):e0209096. doi: 10.1371/journal.pone.0209096 (PMC6296504; doi:10.1371/journal.pone.0209096)

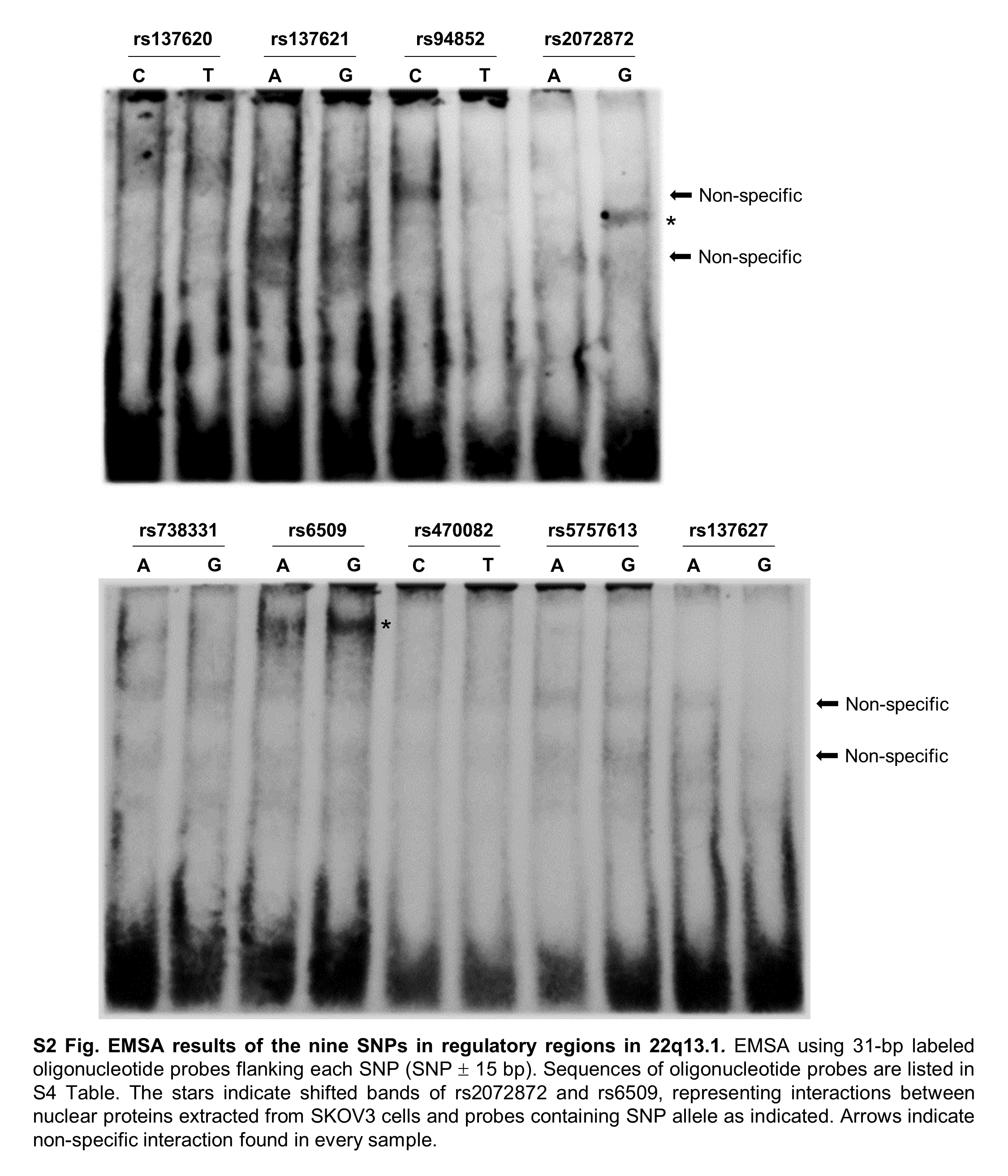

Supplement: S2 Fig — (TIF) [file pone.0209096.s004.tif]
